# Assessment of Groundwater Susceptibility to Non-Point Source Contaminants Using Three-Dimensional Transient Indexes

**DOI:** 10.3390/ijerph15061177

**Published:** 2018-06-05

**Authors:** Yong Zhang, Gary S. Weissmann, Graham E. Fogg, Bingqing Lu, HongGuang Sun, Chunmiao Zheng

**Affiliations:** 1 Department of Engineering Mechanics, Institute of Soft Matter Mechanics, Hohai University, 1 XiKang Road, Nanjing 210098, Jiangsu, China; shg@hhu.edu.cn; 2 Department of Geological Sciences, University of Alabama, Tuscaloosa, AL 35487, USA; blu5@crimson.ua.edu; 3 Department of Earth & Planetary Sciences, MSCO3 2040, University of New Mexico, Albuquerque, MN 87131, USA; weissman@unm.edu; 4 Department of Land, Air, and Water Resources, One Shields Avenue, University of California, Davis, CA 95616, USA; gefogg@ucdavis.edu; 5 School of Environmental Science & Engineering, Southern University of Science and Technology, Shenzhen 518055, Guangdong, China; zhengcm@sustc.edu.cn

**Keywords:** groundwater susceptibility, non-point source, backward travel time probability density

## Abstract

Groundwater susceptibility to non-point source contamination is typically quantified by stable indexes, while groundwater quality evolution (or deterioration globally) can be a long-term process that may last for decades and exhibit strong temporal variations. This study proposes a three-dimensional (3-*d*), transient index map built upon physical models to characterize the complete temporal evolution of deep aquifer susceptibility. For illustration purposes, the previous travel time probability density (BTTPD) approach is extended to assess the 3-*d* deep groundwater susceptibility to non-point source contamination within a sequence stratigraphic framework observed in the Kings River fluvial fan (KRFF) aquifer. The BTTPD, which represents complete age distributions underlying a single groundwater sample in a regional-scale aquifer, is used as a quantitative, transient measure of aquifer susceptibility. The resultant 3-*d* imaging of susceptibility using the simulated BTTPDs in KRFF reveals the strong influence of regional-scale heterogeneity on susceptibility. The regional-scale incised-valley fill deposits increase the susceptibility of aquifers by enhancing rapid downward solute movement and displaying relatively narrow and young age distributions. In contrast, the regional-scale sequence-boundary paleosols within the open-fan deposits “protect” deep aquifers by slowing downward solute movement and displaying a relatively broad and old age distribution. Further comparison of the simulated susceptibility index maps to known contaminant distributions shows that these maps are generally consistent with the high concentration and quick evolution of 1,2-dibromo-3-chloropropane (DBCP) in groundwater around the incised-valley fill since the 1970s’. This application demonstrates that the BTTPDs can be used as quantitative and transient measures of deep aquifer susceptibility to non-point source contamination.

## 1. Introduction

The ultimate goal in most groundwater protection studies is to ascertain groundwater vulnerability, which refers to the likelihood that an aquifer will become contaminated as a function of the contaminant type, source type and distribution, and the system hydrogeology [1,2]. Current index-based popular approaches to assessing the vulnerability of regional-scale aquifers to non-point source contamination rely predominantly on the near surface hydrogeologic information, climate change, land use data, the vadose-zone properties and processes, recharge rates, and/or depth to groundwater [3,4,5,6]. Three groups of methods have been developed to incorporate the above information in the vulnerability map of aquifers, which are the subjective rating method, the statistical method, and the process-based method; see the reviews by the National Research Council (NRC) [1], Gogu et al. [7], and Katyal et al. [8]. Here we briefly review and evaluate these three methods in the following three paragraphs. 

First, the subjective rating (or index) method assigns a numerical score or rating to each (subjective) attribute affecting groundwater vulnerability, without the computational burden of statistically analyzing or numerical modeling of contaminant transport dynamics [8]. This approach has been widely used to map aquifer vulnerability indices, likely due to its relative simplicity and attractive visualization. The most popular and probably the first rating method is the DRASTIC index method developed by Environmental Protection Agency (EPA) [9] and applied and modified by many researchers such as [10,11,12,13,14,15,16,17,18], where “DRASTIC” denotes seven parameters with usually subjective weights to derive the single vulnerability index: Depth to water table, Recharge, Aquifer, Soil, Topography, Impact of the vadose zone, and hydraulic Conductivity. The DRASTIC index motivated many other indicators to map aquifer vulnerability in the last three decades. For example, the SINTACS index modified the DRASTIC index by adding options for the weight rating of each parameter embedded in DRASTIC [19]. The aquifer vulnerability index (AVI) focused on the thickness and hydraulic conductivity of the overlying sedimentary unit. The GALDIT index was originally developed to assess the vulnerability of coastal aquifers [20]. The vulnerability comprehensive assessment index (DI) considered also lithology of vadose zone and the land use pattern [21]. The COP index was built upon flow Concentration, Overlying layers, and Precipitation [22,23]. The subjective rating method is flexible since the basic index, overlay indices, or hybrid ones can all be applied to map aquifer vulnerability. Review and comparison of the above rating/index methods can be found in Abdullah et al. [23] and Luoma et al. [24].

Second, the statistical method uses descriptive statistics or complex regression to analyze contaminant concentrations and spatial distribution, or link observed contamination and environmental factors [25,26]. For example, a logistic regression model was applied by Teso et al. [27] to assess aquifer vulnerability given soil texture data. A Fuzzy rule-based method was developed by Dixon et al. [28] to calculate aquifer vulnerability indices with minimum input of field data. A Bayesian-based methodology was proposed by Worrall and Kolpin [29] and Arthur et al. [30] to assess groundwater vulnerability from single observations of multiple contaminants. Simple statistical methods were also applied to optimize the subjective rating method mentioned above [31,32]. The statistical method sometimes can be very useful since it reduces the data requirements of the other methods (i.e., the index method mentioned above, and the process-based method discussed below); see the review in Pavlis et al. [33].

Third, the process-based method simulates the process of groundwater flow and contaminant transport at the study site, and hence the advantage of this method is the detailed information on the spatiotemporal dynamics of contaminant migration and comprehensive information of groundwater vulnerability. Examples of the process-based method can be found in the recent review of Katyal et al. [8].

As a specific process-based method, the backward travel time probability density (BTTPD) approach was proposed by Fogg et al. [34] to address aquifer susceptibility, and the approach can readily be extended to include the entire vadose zone as well. The method was also used to illustrate the influence of regional- and local-scale subsurface heterogeneity on susceptibility [34]. The BTTPD probabilistically describes the time required for the water and any dissolved contaminants (especially non-point source contaminants) to travel to the sampling location from all possible upgradient source positions (such as the water table in this study); see also [35,36,37,38,39], and it has been applied successfully to determining the release history of contaminants, i.e., [35,40,41,42,43]. Therefore, the BTTPD can be regarded as representing a complete age distribution within a single water sample [44,45,46]. It is also noteworthy that there are intermediate methods between the index and process-based methods, such as the deterministic approach combined with multiple regression analysis proposed by Aschonitis et al. [47] to evaluate the vulnerability of agricultural land to water and nitrogen losses.

Although tremendous efforts have been made to assess groundwater vulnerability, there are two historical challenges. The first challenge is that efforts are needed to assess deep aquifer vulnerability for the following two reasons. First, the indices produced by the rating and statistical methods may only reflect potential of contaminant loading to the water table and do not represent vulnerability of deeper aquifers, especially in highly heterogeneous settings [34]. The working assumption has typically been that the water table represents the top of a “groundwater reservoir,” and that once a contaminant reaches that top surface, the damage is done. In reality, however, most major aquifer systems are thick, aquifer-aquitard complexes within which the travel time from water table to well intakes can easily exceed those within the vadose zone by a factor of 10 to 100. Hence the vulnerability becomes more a function of processes below the water table than above it. Second, the deep aquifer vulnerability issue leads to other important questions related to long-term sustainability of groundwater quality and assimilative capacity of aquifer systems. Clearly, methods that are capable of assessing vulnerability of the entire subsurface are needed.

The second challenge is that previous studies usually quantified the groundwater vulnerability as a static value, while groundwater quality evolution is a long-term process. A transient index may describe better and provide more information about the vulnerability of real-world aquifers.

**Figure 1:**
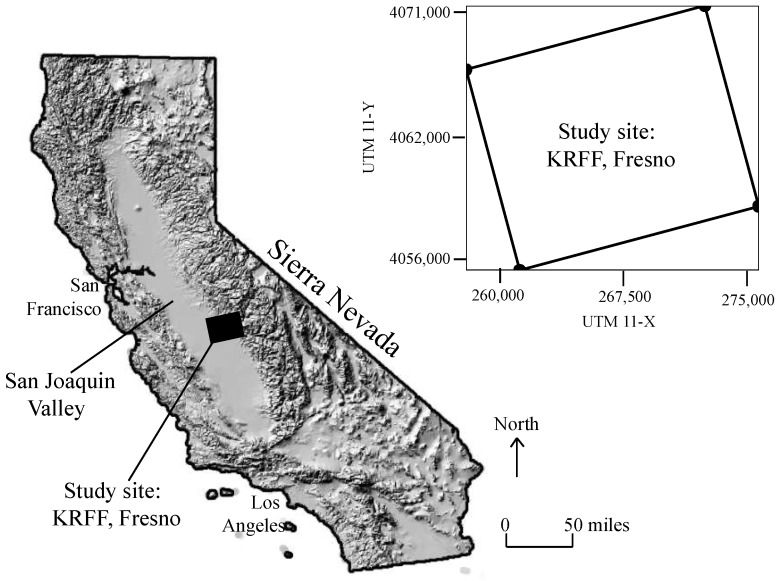
The study area location of KRFF, Fresno, California.

To address the above two challenges, this study selects the process-based BTTPD approach in assessing deep aquifer vulnerability. In the following sections, we will first extend Fogg et al.’s [34] BTTPD approach (see also LaBolle et al. [48,49]) to build the transient vulnerability index for three-dimensional (3-*d*), deep aquifers. To check the feasibility of the transient, 3-*d* index map, we will apply it to assess the vulnerability of Kings River fluvial fan (KRFF) aquifer, located southeast of Fresno, California, USA (Figure 1).

## 2. Methodology Development

Different from previous vulnerability assessment studies, here we address the less restrictive *susceptibility*, which refers to vulnerability to contamination but only as a function of the physical hydrogeology (i.e., independent of land-use and contaminant characteristics) [50]. Thus, conservative contaminants are considered in this study. The method proposed below, however, can be extended to include the effect of chemical and source characteristics on contaminant transport and thus to assess aquifer *vulnerability*, by adjusting the initial/boundary conditions in flow/transport models and/or adding reactive terms in transport dynamics. Clearly, *susceptibility* analysis, or the intrinsic capability for contaminants to intrude on an aquifer system, is a prerequisite for reliable *vulnerability* analysis.

Fogg et al. [34] applied the BTTPD approach to assessing the susceptibility of deep aquifers in the Salinas Valley, using the efficient Lagrangian schemes developed by LaBolle et al. [48,49]. Here we introduce their approach and the research background of BTTPD briefly, and then we explain the extensions we made in this study.

The BTTPD obeys the Chapman-Kolmogorov backward differential equation [51], which has the same form as the backward advection-dispersion equation (ADE):

(1) $$-\frac{\partial }{\partial s}\left[\Theta \;P(X,t|Y,s)\right]=\overset{\leftarrow }{V}\sum_{i}^{}\frac{\partial P(X,t|Y,s)}{\partial Y_{i}}+\sum_{i}^{}\frac{\partial }{\partial Y_{i}}\left[\Theta \;D_{ij}\;\frac{\partial P(X,t|Y,s)}{\partial Y_{j}}\right],$$

where P(X,t|Y,s) is the probability that a particle observed at location *X* at time *t* originates from location *Y* at time *s* (*s* is a time before *t*), Θ is the effective porosity, $\overset{\leftarrow }{V}$ is the reversed Darcy velocity, and *D* is the local hydrodynamic dispersion tensor. Note that here the dispersion tensor has the same value but different meanings in the backward and forward ADEs. In the forward-in-time ADE, *D* accounts for the uncertainty in concentration caused by molecular diffusion and differential advections; whereas in the backward-in-time ADE, *D* accounts for the uncertainty in the initial location and travel time of the particle as it moves backward [52]. The above equation is the adjoint of the forward ADE [36,37,41]. After calculating the velocity vectors for the aquifers, the probability P(X,t|Y,s), or the BTTPD, can be simulated by the backward-in-time random walk particle tracking solutions of the adjoint of the forward ADE [34,38,43], where additional modifications are needed to account for the discrepancy between the forward ADE and its adjoint. For example, the source term in the forward model (where contaminants enter the system) becomes a sink term in the backward model, and the recharge boundary for contaminants (located at the water table) in the forward ADE becomes an absorbing boundary in the backward model [37]. After the BTTPD is solved, the cumulative BTTPD of groundwater younger than a target time can be used as a quantitative measure of aquifer susceptibility.

Three logic extensions of Fogg et al.’s [34] approach are made in this study. First, we produce 3-*d* continuous imaging of aquifer susceptibility instead of Fogg et al.’s [34] discrete susceptibility indexes for separate aquifers located at sparse observation wells. A continuous, 3-*d* susceptibility image can provide whole-system mapping of aquifer susceptibility. It may also help us to distinguish the responses of different subsurface materials, including aquifers and aquitards, to contaminations. Aquitard susceptibility is considered in this study since aquitard materials can form significant sources/sinks of contaminants found in aquifers. Second, the susceptibility of a single aquifer system is treated as a time-dependent transient variable, not the static index calculated by Fogg et al. [34]. A transient susceptibility index can indicate likelihood of aquifer contamination within any time frame. Third, we apply the resultant 3-*d* susceptibility maps to evaluate the influence of subsurface heterogeneity on aquifer susceptibility. This evaluation has not been done by Fogg et al. [34] due to the limited number of aquifers selected for their susceptibility assessment. The subsurface heterogeneity is well known to control the transport of conservative tracers, and thus it plays an important role in generating the physical-process based BTTPDs. Since one of the ultimate purposes of susceptibility assessment is to protect water resources, we need not only to assess aquifer susceptibility, but also to know how the aquifers are contaminated. The three extensions are also the three steps in our approach.

The particle-tracking algorithm used by Fogg et al. [34] needed to be modified to accommodate the above extensions. First, we need to track tremendous (e.g., millions) of particles released from a large number (e.g., thousands) of nodes during one simulation. Preliminary numerical experiments indicated that at least 10,000 particles should be released at each node in the particle-tracking algorithm to get a reliable, relatively smooth and complete age distribution or BTTPD for the node (see also Weissmann et al. [38]). Usually one needs to run one simulation for each node, and it will take weeks to get a 3-*d* susceptibility map for a million-node model in a PC. We circumvent this problem by distinguishing particles based on their releasing locations during one simulation of particle tracking. In other words, particles with labels are simultaneously released from each node and are then back tracked to obtain the BTTPDs (for all nodes) during one simulation. Second, if the node is close to the model boundaries, many particles can exit the model through the lateral or bottom boundaries. The groundwater age refers to the elapsed time since the water enters into the groundwater system through the water table. Therefore, the age is unknown for particles exiting boundaries other than the top boundary (i.e., the water table) in backward particle tracking experiments. In this study, we adjust the ages for these particles based on the depths at which they exit the model as was also proposed by Weissmann et al. [38]. Note that this produces some uncertainty concerning age distributions near the lateral boundaries, and thus the lateral boundary cell ages are less reliable than the internal cell ages.

The backward-in-time method is much more computationally efficient than forward methods, i.e., see [38]. The solutes are simulated to transport backward in time to capture the release history. The calculated age of a particle therefore represents a previous loading-time at the water table. For instance, if the simulation shows a particle in the well is 20 yrs old, this indicates the particle originated from the water table 20 yrs ago and that the capture well will likely be susceptible to contamination 20 yrs after the contaminant migrates downward from the water table. The solutes are also simulated to transport backward in space to estimate their origins.

In addition, because each particle takes on a different path due to the 3-*d* advective flow field and dispersion, each particle history produces a different travel time, therefore generating full distributions of groundwater age within the modeled groundwater “sample”. As will be shown later in this paper, such estimates of complete age distributions are useful and necessary not only for estimating susceptibility, but also for analyzing long-term evolution of groundwater quality as well as the transient susceptibility. The entire age distribution within single water sample generated by the particle tracking method distinguishes this study from other age simulation methods, such as Goode [53], where only the mean ages were calculated. The transient susceptibility proposed here also distinguishes this study from the traditional susceptibility/vulnerability assessment methods reviewed in Section 1, where usually only a static index of susceptibility was used.

## 3. Application to Kings River Fluvial Fan Aquifer

### 3.1. Stratigraphic Sequence and Multi-Scale Heterogeneity in KRFF

We applied the above method to a 190 km^2^ area of the Kings River fluvial fan aquifer (Figure 1). It was selected because the subsurface distribution of geological heterogeneity has already been characterized by Weissmann and Fogg [54] and Weissmann et al. [38,55,56]. The Kings River deposited a large fluvial fan where the river exits the Sierra Nevada into the San Joaquin Valley. Core descriptions show that the aquifer consists of a highly heterogeneous mix of textural and depositional facies, including gravel, sand, muddy sand, mud and paleosol [54,56].

**Figure 2:**
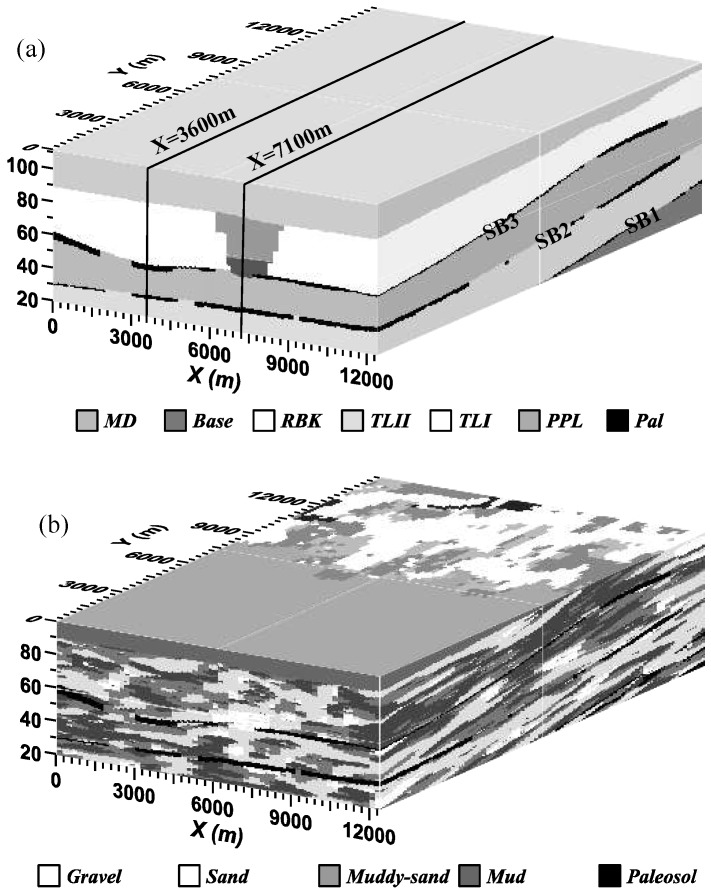
(**a**) Block diagram of sequence distributions in the study area. The SB1, SB2, and SB3 represent the first, second, and the third layer of sequence boundary paleosol. The rectangle within the IVF (*X* = 7100 m) represents the vertical slice shown in Figure 4; and the rectangle outside of the IVF (*X* = 3600 m) represents the vertical slice shown in Figure 5; (**b**) The 3-*d* geological model of the study area.

Five paleosol-bounded stratigraphic sequences were recognized in the study area, dividing the aquifer into discrete zones. In this total section there exist three types of hydrogeologic facies structures that were described by Weissmann et al. [38,56] and are of particular significance for regional contaminant transport (Figure 2). The first structure type consists of laterally extensive, clay-rich paleosols, formed during past interglacial periods. These paleosols are observed at each of the three sequence boundaries (e.g., contacts between the sequences) and are the most laterally extensive confining beds [57]. Other mud-rich facies occur as floodplain deposits, but there are less laterally extensive than the paleosols.

The second structure type exists between each pair of these sequence bounding paleosols and consists of a heterogeneous mix of gravel, sand, muddy sand, and mud of open fan deposits (fluvial sediments deposited across the fan surface during past glacial outwash periods) [56]. Channel deposits in these open fan units radiate outward from an intersection point located northeast of our study area. The heterogeneity in these deposits contributes to contaminant dispersion in the aquifer [38,57].

The third structure type is a coarse-grained incised-valley fill (IVF) deposit identified across the study area [56,57]. This IVF is approximately 3 km wide and 30 m deep, and it is filled with a thick (up to 8 m) coarse gravel (cobble) unit capped by relatively coarse-grained sand.

### 3.2. Modeling Subsurface Heterogeneity, Groundwater Flow, and BTTPD

Hydrofacies within each of the stratigraphic sequences were simulated using transition probability geostatistics, as described by Weissmann and Fogg [54] and Weissmann et al. [38,55,57] (Figure 2b). Observable geologic characteristics, including fining-upward successions, variable orientation of bedding, and major unconformity boundaries between the different depositional units of this fluvial fan are incorporated into the geostatistical results (Figure 2b) [54]. The cell dimensions are 200, 200, and 0.5 m in the depositional strike, depositional dip, and vertical directions, respectively, within a total model domain measuring 12,600, 15,000, and 100.5 m.

Definition of sequence stratigraphy is important for fluvial fan characterizations [58]. Weissmann and Fogg [54] introduced the sequence stratigraphy including paleosols in characterization, but the real significance of it has not been tested much. In order to assess the potential influence of paleosol sequence boundaries on BTTPD and susceptibility, we constructed two different facies models, with and without the paleosol boundaries (models PS and NP, respectively). We built one realization for each model to illustrate the saturated-zone susceptibility assessment approach developed by this study.

Using the geostatistical realizations to generate stochastic hydraulic conductivity (*K*) distributions, we apply the U. S. Geological Survey software MODFLOW [59] to simulate the 3-*d*, steady-state flow field. The conductivities assigned to each hydrofacies were 1.0 × 10^−2^, 1.0 × 10^−3^, 1.0 × 10^−5^, 1.0 × 10^−6^, 1.3 × 10^−7^ m/s for gravel, sand, muddy sand, mud, and paleosol, respectively [38,54,57]. General head boundary conditions with a measured gradient of 0.002 were used in the modeling to simulate inflow and outflow through the lateral and basal boundaries of the model, and the recharge boundary condition was used for the top boundary of the model. The infiltration rate of 150 mm/yr [60] was scaled in proportion with *K* of overlying beds for each cell at the water table [57]. Though significant pumping occurs within the aquifer, thus making the steady state assumption tenuous, we believe this simplified model is appropriate to illustrate the saturated-zone modeling approach. Additionally, even using this assumption, Weissmann et al. [38] were able to reasonably match Chlorofluorocarbon (CFC) concentrations in groundwater, indicating that the steady state assumption may not diminish the overall value of the results. In order to produce susceptibility maps for setting policy, however, we suggest development of models that account for transience, such as pumping and seasonally-variable recharge rates. For the same reason, we suggest generating multiple realizations of aquifers to account for the uncertainty of modeling results caused by the uncertainty of subsurface heterogeneity distributions.

The modeled flow velocities were incorporated into the particle tracking algorithm to simulate the groundwater BTTPD. The same transport parameters in Weissmann et al. [38] were used here, including the longitudinal and transverse dispersivities (0.04 m) (note that the these dispersivities are inconsequential to the numerical results because virtually all of the dispersion is modeled directly through the inclusion of extensive heterogeneity; see also Weissmann et al. [38]), the molecular diffusivity (6.9 × 10^−10^ m^2^/s) and the effective porosity (0.33). The single effective porosity was used for all hydrofacies (assigned with different *K*), since the spatial variation of porosity is relatively smaller than that for *K*, and the impact of the spatial variation of porosity on solute transport is secondary compared with the impact of K on transport [38]. The total modeling time is 200 yrs, with an output interval of 0.1 yr. 10,000 particles were released from the middle point of each node of the model domain to get a reliable and relatively complete BTTPD for each node. Note that the BTTPD of waters located at the middle point of the node is assumed to represent the age distribution at the full node of a size 200 × 200 × 0.5 (*X* × *Y* × *Z*) m. This assumption is based on (1) the conclusion of LaBolle and Fogg [61], who found that the particle tracking solutions are not sensitive to the initial horizontal position of particles; and (2) the conclusion of Weissmann et al. [38], who found that the simulated groundwater ages (especially the mean age) at the middle point of a 0.5 m-long well screen can represent the ages for waters at the whole screen.

## 4. Results

The simulated 3-*d* susceptibilities are shown by the following five slices across the model domains: three horizontal slices at 5, 20, and 40 m below and parallel to the water table, respectively; one vertical slice parallel to the general groundwater flow direction and through (inside and below) the IVF; and another vertical slice parallel to the general groundwater flow direction and outside the IVF (Figure 2a). The three slices parallel to the water table are actually oblique since the water table is not exactly horizontal, but we still call them “horizontal slices” to distinguish them from the other two vertical slices. The 5-m horizon lies above the base of IVF, while the 20- and 40-m horizons lie within and under the IVF, respectively. The vertical slice through the IVF is located in the middle of the valley (Figure 2a: *X* = 7100 m), where the high-*K* IVF truncates most parts of the uppermost paleosol. The vertical slice outside the IVF (Figure 2a: *X* = 3600 m) contains all of the three layers of paleosols, with the uppermost paleosol lying close to the water table.

### 4.1. Susceptibility Maps with Transient Indices Derived from BTTPD

**Figure 3:**
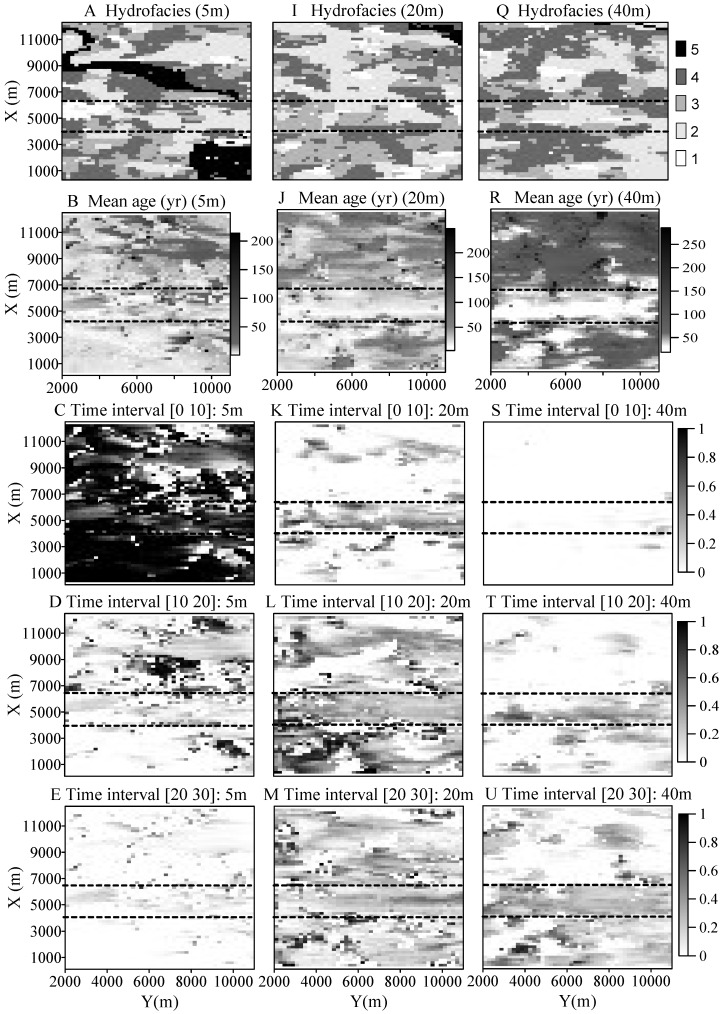

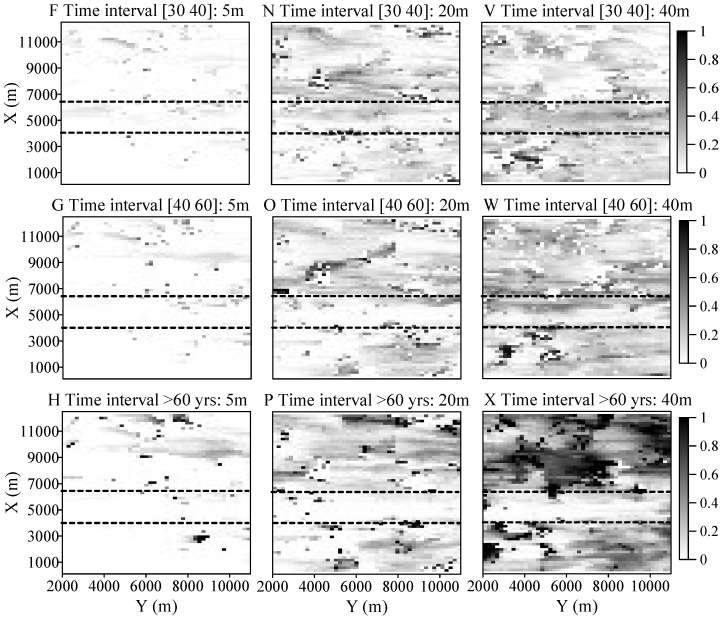
Sensitivity maps for saturated zone 5 m, 20 m, and 40 m below the water table. The areas between the two dashed lines shown in each figure represent the approximated surface areas of the IVF. The left, middle and right columns represent the results for aquifers 5, 20, and 40 m below the water table, respectively. In (**A**) the hydrofacies 1, 2, 3, 4, and 5 represent gravel, sand, muddy-sand, mud and paleosol, respectively; (**B**) shows the mean groundwater age, with a unit year; The cumulative BTTPD within the target time interval [0 10] yrs (**C**); [10 20] yrs (**D**); [20 30] yrs (**E**); [30 40] yrs (**F**); [40 60] yrs (**G**); and >60 yrs (**H**). BTTPD scale is 0 to 1, representing fraction of particles that broke through at the water table.

The susceptibilities along the three horizons (Figure 3) provide an overview of the spatial variation of susceptibilities caused by the multi-scale heterogeneity. First, the simulated mean groundwater ages and cumulative BTTPDs distribute irregularly throughout the area. The zones with young ages do not necessarily correspond to high-*K* hydrofacies since young water can also be found in some low-*K* zones or aquitards. Most of the discrete regions representing old waters, however, generally correspond to the location of aquitards. At a few small, separate high-*K* zones surrounded by low-*K* materials we observe very old water. Second, the IVF appears to significantly influence groundwater age and susceptibility. Inside or underlying the IVF, the system is dominated by relatively young water along the whole valley, independent of the corresponding hydrofacies type. This is especially true for saturated zones 20 and 40 m below the water table, where the shape of valley can be seen clearly from the correspondent susceptibility maps. Outside of the valley, the areas with relatively young water are not well connected horizontally, but intermingled with areas representing very old water.

**Figure 4:**
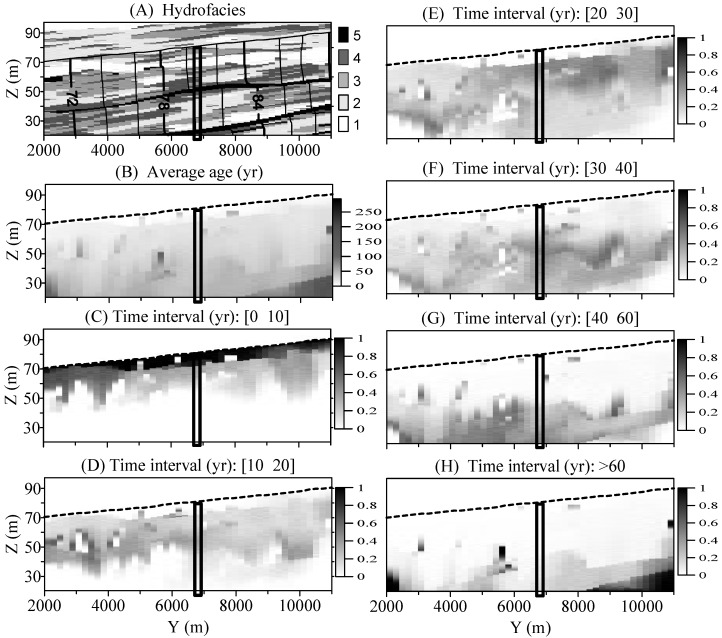
Sensitivity Maps for the vertical slice (*X* = 7100 m) inside the IVF. The dashed line represents the water table. The thick, small rectangle represents the column used in Figure 6A and Figure 7A. In (**A**) the hydrofacies 1, 2, 3, 4, and 5 represent gravel, sand, muddy-sand, mud and paleosol, respectively; The mean groundwater age with a unit year is shown in (**B**); The cumulative BTTPD within the time interval [0 10] yrs (**C**); [10 20] yrs (**D**); [20 30) yrs (**E**); [30 40] yrs (**F**); [40 60] yrs (**G**); and >60 yrs (**H**).

**Figure 5:**
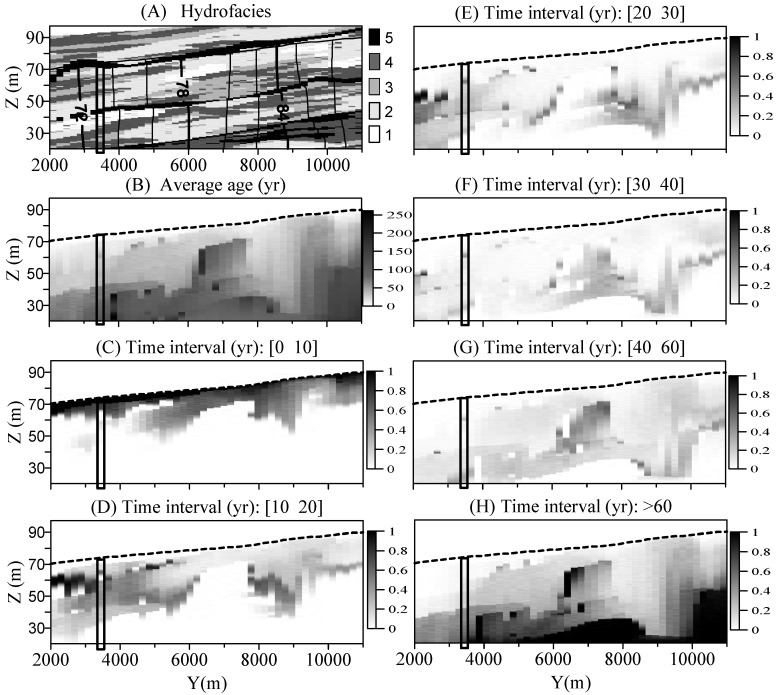
Sensitivity Maps for the vertical slice (*X* = 3600 m) outside of the IVF. Legends are the same as Figure 4. The thick, small rectangle represents the column used in Figure 6B and Figure 7B.

The susceptibility along the two vertical slices basically shows different behaviors from each other (Figure 4 and Figure 5). In the vertical section through the IVF, there are no obvious, preferential paths for the susceptibility “plume” inside or below the IVF. With the increase of backward time, the susceptibility “plume” moves deeper fairly uniformly, and the corresponding mean age generally increases gradually with depth (Figure 4). In the vertical section outside the IVF, however, the susceptibility “plume” moves relatively more irregular. As some places, the paleosol positions are clearly reflected in that the mean groundwater ages below the paleosol boundary tend to be abruptly older than those above the boundary (Figure 5).

### 4.2. The Simulated BTTPD

**Figure 6:**
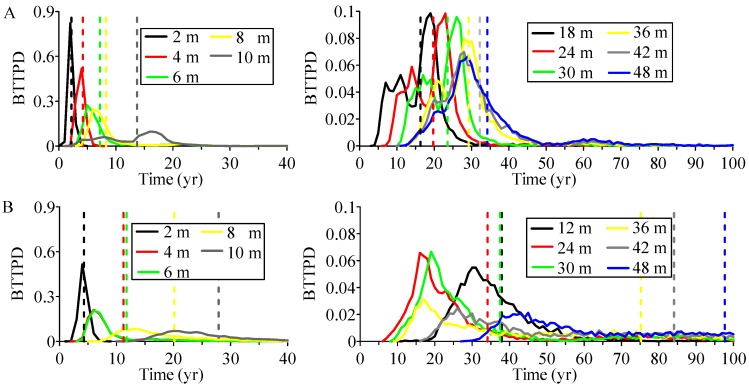
Simulated BTTPD represented by a point at different depths along a column inside of the IVF (**A**) and outside of the IVF (**B**). The column coordinate is *X* = 7100 m, *Y* = 6900 m for (**A**), and *X* = 3600 m, *Y* = 3300 m for (**B**). In the legend, the number represents location (depth below the water table). The dashed line represents the mean age for each BTTPD (same color).

**Figure 7:**
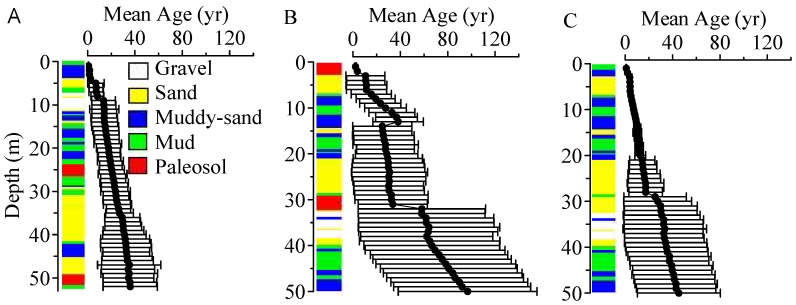
Simulated mean groundwater age and standard deviation along one column inside of the IVF (**A**); outside of the IVF for Model PS (**B**); and outside of the IVF for Model NP (**C**). The column location in (**A**) is the same as Figure 6A. The column location in (**B**,**C**) are the same as Figure 6B.

To further explore the influence of heterogeneity on aquifer susceptibility, we compared the complete BTTPD along two vertical columns inside of the two vertical slices (shown by the small rectangle in Figure 4 and Figure 5). The results are shown in Figure 6 and Figure 7.

For the vertical column (*X* = 7100 m, *Y* = 6900 m) through the IVF, the simulated BTTPD has three main characteristics (Figure 6A). First, the mean water age is close to the mode (which is the BTTPD peak, representing the age with the highest probability) at shallow depths along this column but deviates increasingly from the mode with depth (also see Figure 7A). Second, the spread of age distribution is ≤50 yrs at most locations along the column. Third, the age distribution contains dual peaks at depths below about 8 m. Backward transport simulations show that the particles first move quickly upward, with a small portion of particles exiting the water table directly. This path is the most direct flow path to the water table and it forms the first peak of the BTTPD. With additional time, the remaining particles continue to move further upgradient zones inside the IVF (i.e., to areas located up-fan). They experience longer and more tortuous pathways, forming the relatively large, second peak.

Different behaviors are observed for the BTTPD along the column (*X* = 3600 m, *Y* = 3300 m) outside the IVF (Figure 6B). First, the BTTPD has a much longer late-tail at depths >4 m below the water table. The distribution is highly positively skewed with a single peak, and the mean age is much older than the peak age. Second, the simulated mean age is >30 yrs older than that in high-K IVF deposits (by comparing Figure 7A,B), and an abrupt interface is found at the location of the second deepest paleosol boundary (see the 2nd (deeper) red hydrofacies shown in Figure 7B).To further explore the influence of paleosol sequence boundaries on aquifer susceptibility, we compared the simulated BTTPD’s in Model PS (with paleosol units) to those in Model NP (without paleosol units). Results (by comparison of Figure 7B,C) show that the presence of low-permeable paleosol boundary does enhance the abrupt interface of mean ages, and it also results in a BTTPD with a much older (15~40 yrs older) mean age and a broader spread.

**Figure 8:**
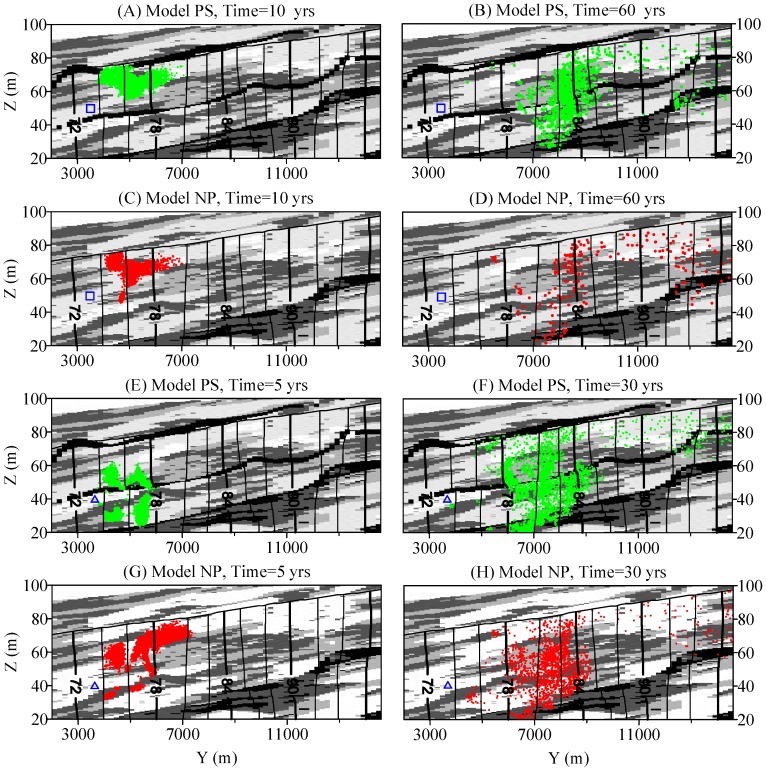
Effect of paleosol boundaries on backward solute transport. Particles releases from hypothetical well screens located above (**A**–**D**) and below (**E**–**H**) the second paleosol layer. A, B, E and F are model PS (with paleosol) results. C, D, G and H are model NP (without paleosols) results. The small blue rectangle in (**A**–**D**), and the small blue triangle in (**E**–**H**) represent the location of the simulated well screen.

**Figure 9:**
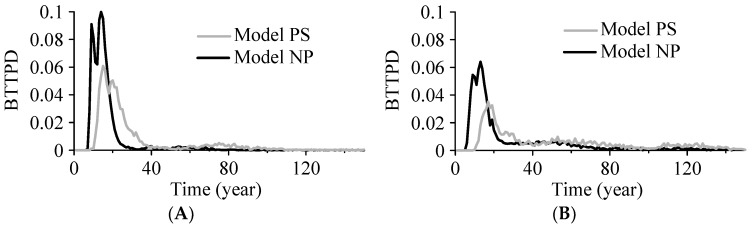
Simulated BTTPD for 2 wells located at different locations in Model PS (the original model with paleosol) and Model NP (the model without paleosol). (**A**) Represents the BTTPD for the well shown by the small rectangle in Figure 8A–D, where the well is above the second paleosol layer; (**B**) represents the BTTPD for the well shown by the small triangle in Figure 8E–H, where the well is below the second paleosol layer.

Additional simulations were conducted to further investigate the influence of paleosol boundaries on BTTPD. Two simulated well screens at different depths were selected, with one well above the 2nd paleosol boundary and the other below the 2nd paleosol boundary (Figure 8). Effective thickness of these screens is 0.5 m. The locations of particle plumes at different backward times were analyzed for both Model PS and Model NP. When paleosol boundaries are present in the model, the particles take longer to reach the water table and more particles will stay in deep, low-K deposits for a longer time before exiting the model domain. This results in a BTTPD with a later arrival peak and a longer late tail (Figure 9). In addition, the mean age at each location along this vertical section in Model PS is larger (by approximately 0. to 50 yrs) than the counterpart in Model NP.

## 5. Discussion

### 5.1. Influence of the Local-Scale Heterogeneity on Aquifer Susceptibility

The local-scale heterogeneity affects the aquifer susceptibility by forming complex 3-*d* heterogeneity structures. The complex relationship between susceptibility and local-scale heterogeneity in the advection-dominated KRFF aquifer system is indicated by the irregular distributions of susceptibilities shown in Figure 3.

Although the influence of local heterogeneity on solute transport has been well-known for decades [62,63,64], the conclusion drawn here is important for aquifer susceptibility assessment- without considering the 3-*d* heterogeneity structure and the transport process of solutes, we cannot reliably relate the aquifer susceptibility to the local hydraulic conductivity and/or the aquifer depth directly. Therefore, caution is needed when using the permeability and depth as the direct indexes of aquifer susceptibility (e.g., less susceptibility for a lower permeable and/or deeper aquifer), which is different from the classical index (i.e., non-physical-process based) method [1].

### 5.2. Influence of the Incised-Valley Fill on Aquifer Susceptibility

The regional-scale IVF significantly increases the susceptibility of aquifers by enhancing rapid downward solute movement. This is because of the ~80% of sandy-materials deposited in this valley. These high permeable materials incise through the uppermost paleosol layer, and pull in higher recharge than other areas in the model domain [38,65]. Therefore, relatively young groundwater with a narrow spread of age distributions can be found inside and below the IVF.

For areas inside and below the IVF, the mean groundwater age may somewhat reflect the aquifer susceptibility, because the mean is fairly close to the mode and also the age distributions are relatively narrow. In addition, the relatively uniform susceptibility within/below the IVF at a same depth is caused by relatively homogeneity of the incised valley fill deposits and lack of extensive aquitard units within it. For aquifers inside and below the IVF, groundwater age usually increases with depth gradually. Hence, the regional-scale IVF has a dominant effect on aquifer susceptibility, overwhelming the influence of local-scale heterogeneity discussed above.

**Figure 10:**
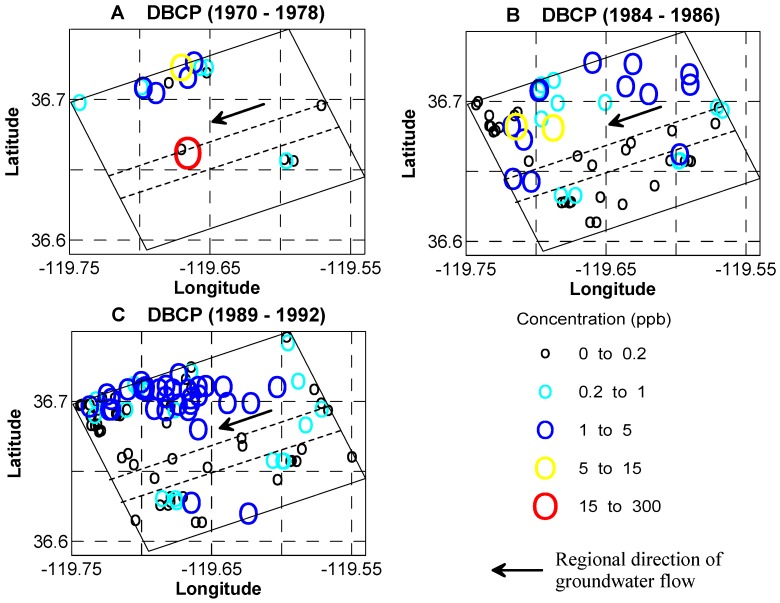
The measured concentration of DBCP during 1970~1978 (**A**); 1984~1986 (**B**); and 1989~1992 (**C**). The rectangle in the middle represents the surface area of the model, and the two short dashed lines give the approximated surface areas of IVF.

The measured concentration of pesticides is used here to test the susceptibility maps, and speculate on the historic role of the IVF on contaminant transport in KRFF aquifer system. 1,2-dibromo-3-chloropropane (DBCP) was a widely used nematicide during the 1950s through the 1970s, with the most frequent use between 1960 and 1979 [60]. DBCP is a persistent pesticide, and it is relatively mobile in soils with high groundwater recharge rates. After leaching to groundwater, the dissolved DBCP moves with water, causing a large area of contamination (i.e., 18,000 km^2^ of DBCP contaminated groundwater in the California Central Valley in 1986 [66]). It was suspended in 1977 and banned in 1979 in California. Since the 1970s (about 15~20 yrs after the first application), the contaminant has been detected in more than 2500 drinking-water wells in California, resulting in a serious nonpoint-source pesticide contamination problem. Considering a non-point source of DBCP contamination, one would expect the most rapid transport to occur in the incised valley fill part of the system. Although historical DBCP concentration data are not adequate to track the DBCP migration through time [66,67], much of the contamination might indeed have intruded deep into the system via the incised valley and migrated laterally toward pumping centers located outside the incised valley. Figure 10 shows available DBCP concentration values for 1970–1978 (Figure 10A, about 20~30 yrs after the first DBCP application), 1984–1986, and 1989–1992. Interestingly, the 1984–1986 and 1989–1992 maps show a preponderance of higher DBCP concentrations in a fairly linear pattern that roughly parallels and lies north-northwest, down hydraulic gradient due to pumping in Fresno, of the incised valley. This is consistent with a scenario in which the DBCP traveled down the incised valley relatively quickly but then was pulled from the valley by pumping for the city of Fresno to the north.

### 5.3. Influence of the Sequence Boundary Paleosol on Aquifer Susceptibility

The regional-scale boundary paleosols within the open-fan deposits affect the BTTPD in two ways: (1) the relatively low permeability of paleosols retards the vertical transport of particles; and (2) the paleosols change the age distribution at a well by bringing more particles from deep zones, where the water age is generally old. This indicates that the overall sequence stratigraphic framework including paleosols decreases the deep aquifer susceptibility to contamination by altering pathways and delaying the vertical transport of contaminants. Therefore, the sequence boundary paleosols have the almost opposite effect on aquifer susceptibility as the IVF does.

The BTTPDs for aquifers affected by the boundary paleosols contain heavier late-time tails than those affected by the IVF, and thus we cannot apply the BTTPD the same way as discussed in the previous section. Especially, for the open fan deposits bounded by paleosols, the mean groundwater-age-based susceptibility estimate will be misleading because the mean groundwater age is significantly influenced by the long late-time tail, missing the peak and the main part of a broad age distribution.

### 5.4. Variations of Susceptibility Assessment between Different Realizations

One could argue that the simulated BTTPDs discussed above based on the single stochastic realization rather than on multiple realizations may not represent the full range of outcomes for transport and susceptibility in this complex aquifer system. Different aquifer realizations may have different distribution properties for both high- and low-permeable materials, resulting in different early-time tail, peak, and late-time tail in BTTPDs. Therefore, the details of BTTPD discussed above, such as the number of peaks and the actual spread length, may vary apparently between multiple aquifer realizations.

However, as indicated by the numerical experiments explored by Weissmann et al. [38], the general behavior of BTTPD will not change significantly among different realizations, if the multiple realizations are built using the same stationary Markov chain model. In KRFF aquifers, the observed high-permeability nature of the IVF will remain in multiple aquifer realizations. Since it increases the aquifer susceptibility more so than any other hydrogeologic feature in the model, the general influence of the IVF on aquifer susceptibility will not change appreciably between different aquifer realizations. Similarly, the laterally continuous and low-permeability nature of the sequence paleosols will also affect the aquifer susceptibility similarly in different aquifer realizations. Another reason the other realizations may not produce very different results is that they all have similar connectivity, which is dominantly determined by the volume fractions. Therefore, although the simulated BTTPDs for local aquifers based on a single realization cannot be used for the purpose of setting policy due to the uncertainty of local-scale heterogeneity, the general influence of regional-scale heterogeneity on aquifer susceptibility may be captured by a single realization, if the Markov chain model captures the main properties of hydrofacies, and if the regional-scale heterogeneity has much less uncertainty (as it should be) than the local-scale heterogeneity. This hypothesis needs to be verified in the future by building multiple aquifer realizations.

## 6. Conclusions

Groundwater vulnerability was usually evaluated by a static index without information on long-term water quality evolution and deep-aquifer response to non-point source contamination. To address this historical issue, this study develops three-dimensional transient maps built upon reliable physical models (with critical geology and hydrogeology information), by extending the backward travel time probability density approach proposed by Fogg et al. [34] and LaBolle et al. [48,49,68], to characterize the complete temporal evolution of deep aquifer susceptibility. For illustration purposes, this approach is applied to assess groundwater susceptibility to non-point source contamination within a sequence stratigraphic framework observed in the Kings River fluvial fan aquifer, California. Three major conclusions are obtained in this study.

First, the BTTPD can be used to derive transient, multi-dimensional indexes of aquifer susceptibility to non-point source contamination, which provides much more useful information than the classical, static index. The BTTPD can, if interpreted appropriately, provide the complete information to decipher the long-term evolution of groundwater quality affected by multi-scale groundwater circulation driven by aquifer/aquitard heterogeneity. Particularly, the cumulative BTTPDs with target time intervals can determine likelihood that an aquifer will be contaminated as a function of space and time.

Second, the regional-scale incised-valley fill deposits increase the susceptibility of aquifers by enhancing rapid downward solute movement and displaying relatively narrow and young age distributions, due to the coarse-grained nature of the IVF and the incision of the uppermost paleosol layer. The historical record of non-point source pollutants (i.e., pesticides) also implied the relatively more rapid downward movement of pollutants, although the data is not adequate to show the detailed evolution of pesticides across the large area of the study site.

Third, the sequence boundary paleosols postpone the contamination of aquifers at all depths by retarding the vertical movement of contaminants and changing the proportions of particle plumes. Therefore, the existence of paleosol boundary decreases the susceptibility of saturated zones to recent contaminants with the price of an increased and a longer-term susceptibility to old contamination.
